# A qualitative study of professional stakeholders’ perceptions about the implementation of a stepped care pain platform for people experiencing chronic widespread pain

**DOI:** 10.1186/s12875-018-0838-y

**Published:** 2018-09-01

**Authors:** Judith Gellatly, Gosia Pelikan, Paul Wilson, Kate Woodward-Nutt, Michael Spence, Anthony Jones, Karina Lovell

**Affiliations:** 1 NIHR CLAHRC Greater Manchester, Division of Nursing, School of Health Sciences, Faculty of Biology, Medicine and Health, The University of Manchester, Manchester Academic Health Science Centre, Manchester, UK; 2 Six Degrees Social Enterprise, Salford, UK; 3 Alliance Manchester Business School, The University of Manchester, Manchester, UK; 4 NIHR CLAHRC Greater Manchester, Salford Royal NHS Foundation Trust, Salford, UK; 5 Human Pain Research Group, Division of Neuroscience and Cognitive Psychology, University of Manchester, Salford Royal NHS Foundation Trust, Salford, UK

**Keywords:** Qualitative, Telephone cognitive behavioural therapy, Stepped care, Implementation, Normalisation process theory, Chronic widespread pain

## Abstract

**Background:**

Chronic widespread pain (CWP) is a major public health problem. Many people experiencing CWP experience mental health problems such as anxiety or depression. Complete relief of skeletal and body pain symptoms is unlikely but with appropriate treatment the impact upon quality of life, functioning and mental health symptoms can be reduced. Cognitive behavioural therapy (CBT) is widely used for a range of health conditions and can have short and long-term improvements in patients with CWP. This research aimed to explore, from a professional stakeholder perspective, the implementation of a local Pain Platform offering a stepped care approach for interventions including telephone delivered CBT (T-CBT).

**Methods:**

Fourteen professional stakeholders holding various roles across primary and secondary care services within the Pain Platform took part in semi-structured interviews. Their views and experiences of the implementation of the Pain Platform were explored. Interviews were recorded, transcribed verbatim and analysed according to Normalisation Process Theory (NPT).

**Results:**

Professional stakeholders were positive about the Pain Platform and its potential to overcome previously identified existing access issues to psychological interventions for CWP patients. It was considered a valuable part of ensuring that patients’ preferences and needs are more readily addressed. In some circumstances, however, introducing psychological interventions to patients was considered challenging and the introduction of new referral processes was raised concerns. To ensure sustainability more work is required to reduce professional isolation and ensure efficient referral procedures between primary and secondary care services are established to reduce concerns over issues related to clinical governance and potential risk to patient.

**Conclusions:**

The findings provide professional insight into the key challenges of introducing a Pain Platform incorporating psychological support across primary and secondary care services within a local service. These included development of sustainable procedures and closer working relationships. Areas requiring future development are identified.

## Background

Chronic widespread pain (CWP), the principal symptom of fibromyalgia, is a major public health problem, affecting between 11 and 16% of the population [1]. It is defined as skeletal and body pain persisting for more than three months and may lack pathologic features [2]. Individuals experiencing CWP frequently experience additional clinical comorbidities such as irritable bowel syndrome, chronic fatigue syndrome, joint pain and headaches [3]. CWP is associated with poor quality of life [4–7] and many people are likely to be experiencing a mental health disorder such as depression, generalised anxiety disorder or panic attacks [8, 9]. In many cases complete relief from symptoms is unlikely but with appropriate treatment the impact upon quality of life, functioning and mental health symptoms can be significantly reduced.

CWP additionally has considerable healthcare economic implications, greater than the majority of other health conditions [10]. Research suggests that CWP is often unsuccessfully identified and managed within primary care and that consequently there is a tendency for overuse of healthcare appointments and thus health service utilisation [11, 12].

The complex etiology of CWP, including its multiple biological, psychological and behaviour elements, presents challenges for its management [13, 14]. While the majority of patients are managed in primary care, in many cases patients with CWP will require access to specialist secondary or tertiary pain services. Primary care practitioners have previously reported inadequacies in the available support, lack of knowledge and understanding about CWP. The need for a multidisciplinary, holistic driven approach has been identified [2, 15–17]. It is therefore vital that primary care practitioners have access to timely and appropriate resources to improve the health and wellbeing of their patients. This includes supporting patients not only with the physical symptoms of their condition but also to enable access to psychological support should they require it.

The Improving Access to Psychological Therapies (IAPT) programme was launched in the UK in 2006. In line with political, social and economic drivers its implementation recognised the major role less intensive psychological therapies could play in the initial stages of a patient’s treatment. Cognitive behaviour therapy (CBT) is widely used for a range of health problems and is effective in enhancing patients’ attitudes and ability to manage their condition. It is frequently provided as part of a stepped care approach, adopted by IAPT services. It is offered in, in low (step two) e.g. guided self-help, computerised CBT (cCBT), and high intensity formats (steps three and four) e.g. longer-term CBT. The lowest intensity CBT-based intervention likely to be effective is offered initially. The impact of the intervention is monitored, and can be stepped up to a higher intensity format or different treatment approach if required.

CBT and exercise has previously been shown to be associated with modest short-term improvements relating to reduction of pain, disability and negative mood in patients with fibromyalgia and/or CWP [18–20]. Recent research with people with CWP found that a short course of telephone delivered CBT (T-CBT) resulted in short term improvement (3 months post treatment) which was sustained in the longer term (24 months) [21, 22]. It was also found to be highly cost effective and has the potential to facilitate faster access to CBT for patients with CWP. While this approach has been shown to be effective, it has not yet been widely adopted as part of routine care, and there are likely a number of factors that will affect its uptake. There is a large literature indicating that efforts at implementing and sustaining new technologies and practices remain problematic [23, 24]. In light of the research evidence, the importance of improving access to such interventions is therefore acknowledged.

**Fig. 1:**
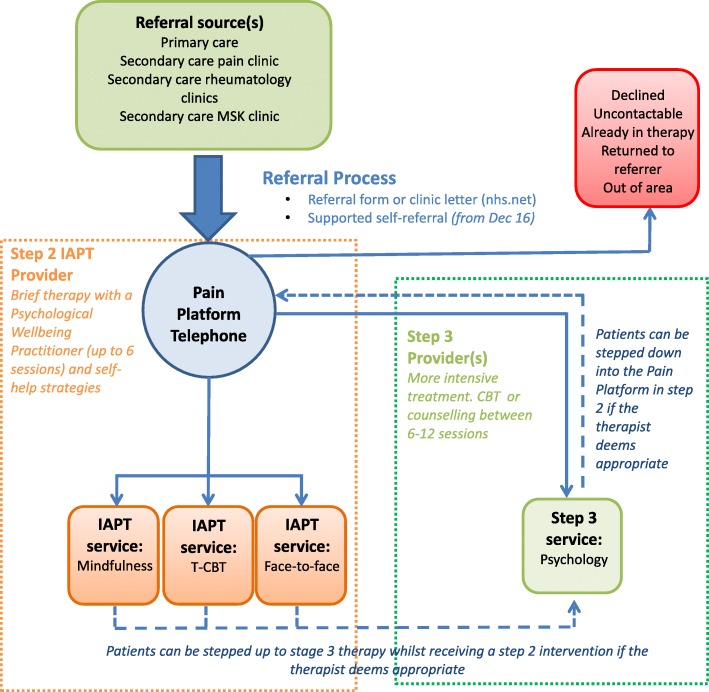
Pain Platform

This paper presents the findings of a qualitative study nested within a feasibility study commissioned by a Clinical Commissioning Group (CCG) that harnessed the opportunity for service innovation by implementing a Pain Platform between primary and secondary care services. The CCG is comprised of a number of neighbourhoods supported by nearly 50 general practices. The Platform, illustrated in Fig. 1, aimed to introduce an accessible route to a pain specific service situated within an IAPT step two service within a Social Enterprise whose aim is to focus on building patient resilience through the delivery of accessible, recovery-oriented services. It provided primary and secondary care referrers with a single point of referral into a range of talking therapies providing a flexible approach to the delivery of evidence based therapies. The Platform provided access into a stepped care approach; triaging referrals and offering a range of interventions including T-CBT and other pain specific interventions or stepping patients up or down to the most appropriate alternative service. Successful implementation of research into the UK National Health Service (NHS) practice requires that new interventions are accepted and welcomed by key stakeholders. This study aimed to explore the narratives of key professional stakeholders including commissioners and managers, referrers, and those delivering T-CBT around the implementation of the Pain Platform alongside existing services for people experiencing CWP over a 9-month referral period.

## Methods

### Theoretical perspective

A qualitative approach was drawn upon to explore the views, expectations and experiences of relevant professional stakeholders of the implementation and sustainability of the Platform and with respect to broader contextual issues regarding the organisation and delivery of care for people with CWP. The views of service users were collated separately by the IAPT provider, as part of service provision rather than research context and are therefore not included here.

A variety of theories exist that can be applied to the understanding of implementation processes, individual and group behaviours [25]. Many incorporate notions of innovation and change via a social influence approach. Normalisation Process Theory (NPT) is a widely used evidence-based theoretical approach that facilitates the understanding of how new interventions become part of existing practices through social organisation of the work and integration into social contexts [26]. In line with its sociological origins, NPT focuses on the ways knowledge is considered, communicated and created within and between groups of individuals at the same time as considering the roles of individual key stakeholders.

NPT is comprised of four main constructs that represent individual and collective levels of work involved in the implementation of new practice:

1. **Coherence:** the sense-making work (meaning) that is conducted by individuals or collective groups of individuals (e.g. organisations) when they are faced with promoting or inhibiting the implementation of a new practice. It focuses on the understanding of what the work is, what it involves and the purpose for which it is being conducted.
2. **Cognitive Participation**: the relational work (commitment/buy-in) that individuals and organisations have to do in order to ensure engagement with the new intervention. It considers how people can contribute, what activities they conduct and what will ensure commitment.
3. **Collective Action**: the operational work (action) that individuals and organisations are required to do to endorse implementation of the new practice. It focuses on how individuals and organisations make it work in practice and what resources are required to promote implementation.
4. **Reflexive Monitoring**: the appraisal (formal and informal) work that individuals and organisations do once implementation has occurred to assess and understand the advantages, disadvantages and impact of the new practice. It considers the ways people assess whether implementation is worthwhile, if improvements can be made and sustainability.

NPT is therefore useful for this particular study, providing a robust analytic framework to assist with the exploration of the implementation of the Pain Platform. We aimed to capture the views of as wide a variety of professional stakeholders as possible including those referring patients to the service, practitioners supporting patients in the service and those commissioning/managing the service. Participants were therefore recruited via purposive sampling [27] from professionals who were working across the Pain Platform. Snowball sampling where existing professional participants assisted with the recruitment of relevant colleagues/acquaintances also took place. This involved those commissioning, referring to, managing and delivering interventions within the existing and newly implemented Pain Platform.

Individual interviews were conducted 3–7 months after commencement of the Platform to ensure that initial embedding had occurred and communication of the opportunity to refer had been established among appropriate services. To ensure consistency across interviews, a topic guide incorporating key topics and open-ended questions linked to the study aims and conceptual ideas of NPT was developed. The topic guide included questions to elicit participants’ perceptions and experiences of implementing and delivery of treatments within the Pain Platform and how it compared to pre-existing process of care delivery. The opportunity was also offered to participants to make any additional comments about issues relating to implementation of the Platform.

One member of the research team, not directly involved with any of the referral or delivery services, conducted all interviews face-to-face or by telephone, dependent on participant preference. All participants provided written consent which included consent to audio record the interview. Interviews lasted from 24 to 71 min and were transcribed verbatim. Data was managed using NVivo software [28] and independently analysed by the researcher who conducted the interviews with the assistance from a Psychological Wellbeing Practitioner (PWP) who had supported patients accessing T-CBT using thematic analysis [29] informed by NPT. Using aspects of the constant comparative method of analysis [30] data was coded and explored to identify categories and their relationships with one another across interviews. Following this the categories were mapped on to the NPT framework [26].

## Results

Fourteen individuals took part, 7 females and 7 males. Participants included 7 service providers (PWPs, operational/service managers and supervisors); 6 referrers (general practitioners rheumatology and physiotherapy practitioners) and 1 commissioner.

Emergent themes were mapped into the NPT framework. All themes were accounted for within the framework. The themes are presented in Table 1 and are structured around the four key constructs.

**Table 1:** Study findings presented using NPT core constructs

NPT construct	Study themes
Coherence	- The Pain Platform has the potential to overcome existing access issues
	- The Pain Platform incorporates shift towards parity of esteem (valuing mental health equally with physical health)
	- The Plain Platform is advantageous for patient access and need
	- The Pain Platform demands an increase in professional understanding/awareness
Cognitive participation	- Professionals working in the Pain Platform will need to foster patient engagement
	- Availability of the Pain Platform is valued
	- Front-line support of the Pain Platform may be variable
	- Platform user acceptability of the Pain Platform influenced by personal beliefs
	- The Pain Platform is aligned with existing platform procedures
Collective Action	- The Pain Platform implementation requires addressing professional beliefs about CWP
	- Mixed delivery methods may be required for the CWP T-CBT
	- The Pain Platform is aligned with existing platform goals
	- Pain Platform implementation requires additional resources
	- Pain Platform implementation requires alignment with existing referral protocols
Reflexive Monitoring	- Feedback for professionals regarding impact of the Pain Platform will enhance learning
	- Need to enhance opportunities to foster good collaborative care to ensure success of the Pain Platform
	- The Pain Platform’s success and sustainability is reliant on further development of the organisational infrastructure
	- The Pain Platform’s success is reliant on practitioner training and support
	- Pain Platform referral protocols need to align with existing platform procedures

### Coherence: Understanding and making sense of the pain platform

Implementing new approaches to the management of any condition relies on individual workforce members collaboratively developing an understanding of the new practice and its potential value. Understandings of access routes to current psychological therapies for people experiencing CWP were at times misinformed. Despite the opportunity for GPs to refer directly to the IAPT service for mental health problems some regarded the referral process as an indirect and potentially lengthy process, resulting in patients not receiving the support they required at the point that mental health problems became a concern:

“*I work in a GP practice…at the moment, the only way of me getting any form of psychology, or interventions which are tailored to patients with pain, is to refer them to the hospital, and they will go to the pain clinic, they’ll be assessed there and then and if they are deemed to be appropriate for psychological interventions then they’ll have their sessions. So that can take a few months*…” (030, Commissioner)

While some participants, mostly GPs, identified barriers to access, in contrast others voiced their opinions of the Pain Platform in a positive light, recognising the potential it had to overcome such access issues providing timely and appropriate support for patients when required. The importance of timely support was voiced by many with reference to existing unhelpful pathways to psychological care being drawn upon. There was a general consensus that the current journey that participants took to get appropriate psychological care was lengthy and often unsuccessful with some service users “*just stuck in no man’s land*…” (008, Commissioner).

The new Pain Platform was identified as not only offering a quicker response but also opening up new opportunities for improving patient acceptability, predictability, choice and subsequently their overall experience:

“*mostly I think they [patients] really like it [being referred to the service]. They like the idea of something happening reasonably quickly, because they’re just used to being fobbed off from one thing to another, one consultant to another, one service to another…that might have been over two to five years sometimes and then finally they get somewhere where they can actually get the therapy that they need…that’s a disaster really. So this is [an] improvement. So I think it’s brilliant*.” (025, Referrer)

Implementation of the Pain platform was also regarded as a way to address the need to achieve parity between mental and physical health, aligning services with national policy initiatives [31] and guidance that recommends incorporation of psychological support for the management of CWP. In drawing upon their experiences of the limited benefits that one treatment approach in isolation can have on patient outcomes, participants recognised the value for the adoption of a holistic care approach. :

“*It was a missing piece of the jigsaw [the CWP service]… I think as a patient living with a chronic pain condition no matter what the psychological support is, you need an outlet to be able to moan essentially. Even if it’s counselling or something you need some kind of support from that level – possibly even with a normal HADS [Hospital Anxiety and Depression Scale] scale score. Certainly for those patients that score higher and therefore it’s NICE guidance recommend I think is an essential part of the service really*.” (002, Referrer)

Despite views regarding implementation of the Platform being positive, several participants deliberated upon the diverse levels of understanding of CWP presentations by referring professionals. The impact these variations may have upon their engagement with the Platform was recognised. Given the lack of guidance within services, professionals often work in isolation and develop their own ‘systems’. Linking with previously discussed misunderstandings about referral processes, one GP acknowledged that as a result of continuous service developments and changes the new Platform may be underutilised as a result of practitioners not being confident in its suitability or unaware of its purpose or existence.

“*Services for GPs, availability of services come and go like fashions and we cannot keep up-to-date. If you think that across every service that we refer into, whether that’s social services, occupational therapy, community physiotherapy, community dietetics, physios, social aids, psychology, for each individual person working within that area that is their priority. For us it might be one of 50 and I can’t keep up-to-date with the minutia, that the referral form has changed or the destination has changed or the fax number has changed or there’s been a slight tweak in criteria. So if you want people to be referred into the service we need to know the service is there but just to be told that you can refer on to the back of your normal primary care psychology service referral is really good*.” (029, Referrer)

### Cognitive participation: Professional engagement with the platform

Implementing new models of health care provision is reliant, in part, on changes to existing systems, procedures and conduct. The participation of individual stakeholders affected by such changes is paramount and is influenced by the extent to which their participation is promoted and how committed they are to the new ways of working.

The importance of the new Pain Platform was acknowledged by all participants and was regarded as being aligned with existing working practices. Some drew upon the concept of ‘parity of esteem’ within mental health care, which in essence considers the need to value mental health equally with physical health. Some participants recognised the need to take a more considered approach towards the management of mental health problems within physical healthcare systems:

“…*when we think of physical health conditions the way we probably need to move and the direction we need to go in is to start to develop things in a way where further down the line we have wherever it’s appropriate psychological intervention going on as a core part of a service. Rather than everything being seen as it should be referred to specialist mental health services and seeing psychological wellbeing as more of a core thing that’s addressed and provided for within services*.” (008, Commissioner)

Some stakeholders, however, recognised that the move towards giving apriority to mental health in line with physical health had not been accepted fully across all services and was identification of the potential variability of commitment by all parties:

“*I think there are a few dimensions to this, so one is attitudinal, so the decision makers be they very senior managers or executives within a health provider trust…whether those are commissioners whoever they are, if we look at the NHS for example, this whole thing that’s been talked about more and more just in recent years of Parity of Esteem, I wonder how much that is really thought about in planning generally and taken seriously enough. We’re starting to get there but I think there’s a lot of work to be done*.” (008, Commissioner)

One individual recognised that in order to ensure investment, incentives for front-line stakeholders may be required:

“*My experience of engaging with GPs en masse in [local site], is that it’s very difficult and even the GP leads say they find it difficult. So I think there would have to be some kind of incentive to do that and I’m not quite sure what that is*.” (025, Referrer)

### Collective action: Implementing the platform into practice

Collective action is the work that individuals need to perform in order to implement new procedures and practices. It takes into account how people interact with others, the emerging skills, knowledge and confidence developed over time, allocation of roles and tasks and resource availability and usage. Stakeholders were initially optimistic about the implementation of the Pain Platform, however over time several participants identified and reflected on potential barriers to its integration and sustainability.

While the Platform was widely regarded for its ability to address access issues, many stakeholders had concerns about the delivery of the intervention via telephone. Some lacked insight into the approach and reported negative views towards its use. In comparison to traditional face-to-face approaches it was sometimes regarded as a barrier to patient engagement and Platform success:

“*I am probably a bit sceptical about the phone. I think that is due to that you lose body language cues and facial cues. I think in terms of all that we do in consulting I think the telephone is sometimes far from ideal. You don’t know just how much of the attention you’ve got of the person on the other end. Like I’m sat talking to you today and I can tell you I’m sat on my sofa with a cup of tea but I could be well be sat reading my work computer or flicking through Facebook or doing all sorts of things and you wouldn’t be aware. So I think that for me and I also think that in terms of communication is that you don’t necessarily…can react if you’ve said something that somebody doesn’t seem happy with, especially when you’re exploring that notion that somebody’s physical symptoms may have a psychological grounding that I think for some people they may get offended by that*.” (029, Referrer)

For those involved in the delivery of the Pain Platform it was thought that some patients were perhaps less invested in using the telephone as a means of receiving therapy and that this could impact upon division of labour and resources:

“…*trying to get hold of those patients is quite hard, ringing them multiple times and not being able to get through, getting through and then booking them in for an appointment, and then because it’s a telephone appointment I’ve noticed that a lot of people don’t have the same mind-set about the telephone appointment… someone’s popped round for a brew, or they’ve gone to Tesco’s and forgotten, and they can’t speak… So then you rearrange the appointment and the same thing happens again… So you might have had four telephone contacts with somebody before you’ve actually even done an initial assessment with them. Which then obviously means that that time is taken up… So you can’t take somebody new in because they’re taking up a space, but then they’re not actually doing anything in that space… So it seems like people don’t see it as important as a face-to-face or as rigid as a face-to-face maybe, maybe it’s not important, maybe it’s that kind of they feel it’s more flexible because it’s on the phone…*” (016, Service Provider)

Investment in communicating the purpose of the Platform to patients was an additional threat to patient engagement and a cause of uncertainty. Given that the primary symptoms experienced by CWP patients are physical in nature, a psychological treatment option is not principally offered. The incorporation of the service caused concern for some participants who did not feel confident about how it would be perceived by patients and their ability to communicate its purpose in an effective and timely manner.

“*There are patients who take a while to understand that pain is an integrated experience, so then it’s a matter of choosing the time for the intervention. So I think timing is very important. So that’s why I think it’s quite important to have a relatively unburdened service, which is not the case for my service at the moment, because you want to be able to, you know, bring in some kind of talking therapy at the time when they’re most ready to accept it and engage with it. That needs a bit of fine tuning in terms of service*.” (025, Referrer)

Furthermore, issues with the Pain Platform referral protocols caused concern for some with one referrer indicating that they were “*terrible*” (002, Referrer). Difficulties obtaining access to NHS email accounts required to securely send electronic referrals to the service was recurrently identified as a barrier, and differed to usual postal procedures. Some thought that adaptation to referral procedures deterred some from referring:

*“…I don’t think they’ve referred anyone yet [secondary care musculosketal (MSK) clinic local clinical pain team] – and I can understand that when they are used to referrals being easier. It seems silly that we need to make it as simple as possible for the referrers, but it will get more work through [patients referred] the more simple it is.*” (002, Referrer).

Others didn’t feel a change to existing referral procedures was warranted:

“*we just send a copy [of the referral]. So we dictate the letter as normal, dear sir, can you see this patient? But I believe that some of those get [e-] mailed rather than posted…I suppose it is a bit strange really, ‘cause all of the other communications, which should have the same degree of confidentiality, aren’t being handled in a different way.*” (014, Referrer)

### Reflexive monitoring: Appraising the platform and future directions

Participants deliberated upon the implementation of the Pain Platform reflecting upon existing pathways in their thoughts about future sustainability and benefits. As many were unclear of the expected benefits for patients, attention shifted to the value of feedback and its importance in optimising patient care and service operationalisation. Given the short implementation period many had not had the opportunity for direct patient feedback.

“*I don’t want to be referring in forever without knowing that it’s useful. And particularly for a condition where I may not follow people up. So, if I see somebody with a new diagnosis of fibromyalgia where I am kind of confident that that’s the diagnosis, I can review some results afterwards and know that they haven’t got any other problems. I think that I have referred them into a service that is going to help them, but then never know whether that has been useful over many years.*” (001, Referrer)

“*linked closely and sitting right behind that [clinical outcomes] is patient experience because I guess no matter how effective the intervention...the people who are delivering it, the style of how it’s delivered, where it’s delivered et cetera the patient will have views on that and will interpret it in terms of their own personal experience. And if it wasn’t a good one for them in other ways then they won’t want to tap into that much in the future. So the whole patient experience thing is very, very important*.” (001, Referrer)

Working under the auspice of collaborative care between the Pain Platform provider and the main referring services, such as the secondary care MSK services was considered as the most appropriate way to optimise the implementation of the Platform. Lack of collaboration in the way in which the Platform was currently implemented was identified as a barrier. Establishing links between physical and psychological services was viewed as important to maximise continuity of care and has the potential to improve patient experience. This related to the locality of services, building relationships and sharing ideas and knowledge between different services. For some the co-location of services within the same or nearby buildings was highly beneficial.

“*Certainly when you are working with the service you are referring into, if you know the person, if you see them regularly or bump into them or whatever that does make it a much smoother job which hasn’t happened with this understandably.*” (002, Referrer)

“*You have a worker that is sort of embedded within the team [in other comparable services], so they have close links in one way or another. Whether that’s going in every now and then and meeting with the team. With the diabetes team, we used to have a clinic room alongside the clinical rooms, out in the community, so that the team could, say, even just if you’ve not got that many patients, just knock on your door and ask you a question… the other thing that I found it quite useful for, being co-located, is just that relationship with the referrer. So the referrers often they’ll ring here just to run a case by us or they’ll say, do you want to come to my team meeting and just do a quick catch-up of where we’re at? So there’s something about that co-working that I think helps that relationship, and as well then they get a bit of feedback where this patient has gone and whether it is helping, you know, overall and…*” (020, Service Provider)

“*Making sure that there is good links between all the different services so it doesn’t just become a siloed thing…*” (034, Service Provider)

The sustainability of the Platform and the demand for more efficient referral procedures to be implemented were frequently discussed. Current procedures that did not fit with existing referral protocols were of concern. Reducing the amount of additional work that is required was viewed as important to ensure efficiency.

“*We probably need some kind of online referral process, a button we press ideally…if you’ve got a clinic, everything’s in the clinic letter, press the button, that’s all you need to do. That would be the ideal thing. Not repeating everything again*.” (025, Referrer)

The success of the Pain Platform was also reliant on continued awareness of practitioner training needs and availability of CWP-specific training. Without the necessary skilled workforce, operational challenges were anticipated.

“… *it’s like any, sort of, development, it’s what skills are required... do practices need some extra development, extra skills, extra training etc. to deliver that. If it is the latter then I think that becomes more problematic because what then tends to happen is you don’t go overnight to a whole team being trained. So, it tends to develop in dribs and drabs if you like... What you don’t want to do is to have obstacles*… *so if we take chronic widespread pain, if that condition can be worked with step two practitioners, step three practitioners with a core qualification like CBT, then that doesn’t present that challenge. It’s when you determine that actually somebody needs an additional level of skill to deliver that or qualification to deliver that, that you then get operational challenges.”* (033, Service Provider)

In acknowledging that low-intensity psychological services commonly have a high turnover of staff the need for ongoing access to training and supervision to ensure an adequately trained and supported workforce is in place was considered vital.

*“…it [service model] needs to be backed up with training and supervision… Because if you just provide training and you don’t provide the supervision and support, then it, just like, dies a death*.” (018, Service Provider)

## Discussion

The Pain Pathway was valued as an approach offering the opportunity better manage and meet the needs of people experiencing CWP. In exploring key stakeholder perspectives of the implementation of the Platform, from individual and organisational levels, it has been possible to obtain evidence regarding the probable barriers and facilitators faced. Implementing new service innovations is necessary on a number of levels. Any changes to services need to be focussed not only on health service improvement and also on demonstrating patient benefit. Whilst clinical and applied health research explores health and economic outcomes less emphasis is often placed on the use of evidence-based frameworks to establish the likelihood of success of implementation into routine practice, or indeed the potential implications of implementation ability on those outcomes.

Analysis informed by NPT has elicited an understanding of the ways in which the Pain Platform was welcomed by those involved in its referral, delivery and management. It also has provided a means for enhancing understanding of the ways in which the Pain Platform can be modified to best meet the needs of the workforce and ensure sustainability.

The Pain Platform aimed to improve the management of CWP, in part improving access for patients to effective psychological treatments. Psychological factors have been found to contribute to predictors of poor outcomes in individuals experiencing CWP [32, 33]. Therefore offering an accessible opportunity to explore such factors within the Pain Platform was regarded as beneficial by most stakeholders with the potential to improve outcomes. In doing so healthcare professionals identified the difficulties sometimes faced when exploring this new treatment option with their patients. The need to understand the impact of CWP from a patient’s perspective was important but challenges were sometimes faced when offering a psychological intervention when the problem was physical, possibly conveying to patients that they have a lack of understanding of their symptoms. These findings are consistent with previous literature that highlights that some patients feel their pain is not acknowledged and considered to be ‘all in the mind’ [34, 35]. It has also been acknowledged that many healthcare professionals, specifically GPs, experience difficulties managing patients experiencing pain [36]. Primary care practitioners and GPs have concerns regarding inadequate pain management and recognise the need for educational initiatives to be embedded within their working practice. [37, 38]. Similarly, patients have expressed the need for GPs to be better educated about CWP and improve their communication skills [39]. Clinicians need to be able to effectively communicate the benefits of integrated approaches to pain management, including psychological support.Using NPT as a conceptual framework assisted in exploring the implementation process and uncovered interpersonal organisational relationships that require further work. This is consistent with existing ilterature which highlights four often interdependent barriers and enablers to implementation; namely the context in which implementation takes place; the influence of organisational features; the characteristics of healthcare professionals involved and the characteristics of the service change itself. [40]. Within this study it was suggested that barriers to success related predominantly to the influence of organisational features that hindered the development of efficient and secure referral processes and inter-professional communications.

Collaborative care is promoted within healthcare services as a means to work efficiently and ensure patient needs and preferences are met [41]. Previous literature has highlighted a desire among GPs for a collaborative approach in the management of CWP [42] which was mirrored by the professionals in this study. However, professionals working across the Pain Platform reported that collaboration was difficult. This was primarily due to poor accessibility related to the provider and referrers not being co-located. Integration of services and workforce members across services was central to views upon the success of implementation. Poor integration, resulting in professional isolation, was identified as a key factor related to communication difficulties between provider and referrers and raised concerns over risk and governance for the provider.

Stakeholders identified that patients with CWP may be distressed about a variety of life events. No clear pathway/ethos to manage this was established and it was dependent on individual professional clinical judgement/ reasoning. Development of a collaborative working approach, with a shared sense of purpose and agreement, is therefore vital to assist with the understanding of patients’ psychological and emotional journey through the Platform.

Professional stakeholders had divergent views and perceptions of the Pain Platform and its success which is as expected given their different roles. Beliefs and attitudes towards the implementation of a T-CBT intervention may have influenced perceptions around sustainability and success. Beliefs regarding the use of the telephone as a treatment modality and reluctance towards changing existing referral procedures may need to be addressed to ensure the commitment of all individuals within the Pain Platform.

Professionals reported some of the administrative processes, particularly electronic referral processes, were not consistent with current procedures for some services. This was reported to have a negative impact on predominantly secondary care administrative staff where the possibility of increased workload or adopting a less-convenient referral approach was challenging. Although a short-term solution was established during the project, involving one research team member hand-delivering referrals, it was not sustainable and no alternative was found that was acceptable to both provider and referrer. Greater flexibility of working practices such as having the capacity and ability to adapt to changing procedures and circumstances may assist in overcoming this barrier to sustainability.

Despite successful implementation of the T-CBT intervention further development of the organisational infrastructure across primary and secondary care services to support the Pain Platform was considered vital by the service provider of the T-CBT intervention. They raised concerns over the maturity and sustainability of the current working model. It was believed that without the support of the research team that it would be challenging to continue supporting patients via the Pain Platform without adaptation due to concerns over issues related to clinical governance and risk to the patient. Additional work to increase knowledge and awareness of the pathway among referring services/individuals and improving the integration of services involved would be beneficial. T-CBT will still be made available for patients with CWP who are referred into the IAPT provider via the usual/traditional GP referral processes that exist; receiving T-CBT will be based on individual preference and clinical need. However, more work would is required to establish a fully integrated pan-organisational Pain Platform that can be operationalised in a sustainable way.

### Limitations and strengths

The study time restrictions meant interviews were conducted relatively soon after implementation had occurred and on one occasion only. Therefore the data captured may reflect only initial experiences of the Pain Platform. Future work exploring the experiences and views of implementation could include studies of a longitudinal design to better capture if perceptions altered over time.

Although professional stakeholders varied in terms of their role within the Pain Platform it is acknowledged that the sample is restricted to one locality and is relatively small which could limit generalisability to other pain management settings. Participants provided representation for the key routes through the Pathway, however, as the Pathway was early in conception/implementation, routes were developing and to some extent limited. In addition, only the perspectives of professionals are represented thus patient perspectives are lacking within a research context. It would be of value to explore if patients regarded the service as valuable, whether organisational issues experienced by professionals impacted upon or influenced the experiences and perceptions of patients and if they would advocate any adaptations to the Pain Platform. Despite a small sample, it is important to acknowledge the majority of professionals interviewed were independent of individuals and services that were involved in the development and implementation of the Pain Platform. Thus they were able to offer non-biased feedback and viewpoints on the sustainability and sustainability.

## Conclusions

Despite the Pain Pathway being valued, the findings provide insight into some of the key challenges and areas for development in implementing and sustaining a Pain Platform for psychological support within a local service. The introduction of new referral processes was not wholly welcomed and difficulties introducing new interventions to patients highlighted.The importance of a sharing a common ethos and workable referral process between services and referrers to ensure that professional stakeholders can manage referrals and support the psychological needs of patients effectively is vital.

## Abbreviations

CBT: Cognitive Behaviour Therapy
CWP: Chronic Widespread Pain
GP: General Practitioner
HADS: Hospital Anxiety and Depression Scale
HRA: Health Research Authority
IAPT: Improving Access to Psychological Therapy
MSK: Musculoskeletal
NHS: National Health Service
NICE: National Institute for Clinical Excellence
NPT: Normalisation Process Theory
PWP: Psychological Wellbeing Practitioner
REC: Research Ethics Committee
T-CBT: Telephone-Cognitive Behaviour Therapy
